# Bibliometric Analysis Study on the Mechanisms of Brain Energy Metabolism Disorders in Alzheimer's Disease From 2000 to 2020

**DOI:** 10.3389/fneur.2021.670220

**Published:** 2021-07-21

**Authors:** Yi-Hong Du, Ruo-Yu Yang, Qi Wang, Li-Yan Wang, Lei-Chao Liang, Lei Zhu, Yan Sun, Ming Cai

**Affiliations:** ^1^Shanghai University of Medicine and Health Sciences Affiliated Zhoupu Hospital, Shanghai, China; ^2^College of Physical and Health, East China Jiaotong University, Nanchang, China; ^3^College of Rehabilitation Science, Shanghai University of Medicine and Health Sciences, Shanghai, China; ^4^College of Sport Sciences, Qufu Normal University, Qufu, China

**Keywords:** Alzheimer's disease, brain energy metabolism, bibliometric analysis, Web of Science, CiteSpace, VOSviewer

## Abstract

Alzheimer's disease (AD) is currently one of the main diseases afflicting the elderly in the world. In recent years, more and more studies have shown that brain energy metabolism disorders are the key pathogenic factors and main early pathological features of AD. Many risk factors such as insulin resistance, mitochondrial dysfunction, oxidative stress, Aβ-amyloid plaques, neurofibrillary tangles of hyperphosphorylated tau, aging, and neuroinflammation are involved in brain energy metabolism disorders. In this study, 1,379 Web of Science publications on the mechanisms of brain energy metabolism disorders in AD, all published from 2000 to 2020, were analyzed. Some network maps were drawn using CiteSpace and VOSviewer software which can be used to clarify research focus, forecast research frontiers and development trends, and provide different perspectives and characteristics in AD brain energy metabolism disorder mechanisms.

## Introduction

In recent years, dementia has become a major risk factor in the daily life of the elderly. The worldwide prevalence of dementia is predicted to almost double every 20 years, from 46.8 million in 2015 to 131.5 million in 2050. Among dementia, nearly 3/4 are AD patients (1). Energy metabolism disorders have become a popular direction in the field of prevention and treatment of AD. Some clinical research on positron emission tomography (PET) scans have shown that the reduction of brain energy metabolism precedes the overt symptoms of AD by decades (2, 3). The key brain fuel is glucose, almost 25% of the total body glucose is used by the brain, much of which is used to sustain synaptic transmission and support neuronal function (4). Brain glucose metabolism includes a glucose uptake mechanism such as insulin or the insulin signaling pathways that control brain glucose uptake and then glucose transporters (GLUTs) transporting glucose into the brain. Glucose in the brain produces adenosine triphosphate (ATP) *via* a series of processes of glycolysis, tricarboxylic acid cycle, and mitochondrial oxidative phosphorylation (OXPHOS) in the mitochondria. Overall, multiple signal pathways, transporters, and metabolic enzymes operate in combination with mitochondria to maintain sufficient fuel supply and energy storage to maintain neuronal activity.

Aβ-amyloid (Aβ) plaques, neurofibrillary tangles of hyperphosphorylated tau, aging, and neuroinflammation are all risk factors for glucose metabolism disorders. These risk factors may lead to glucose transport and metabolism disorders, insulin resistance, mitochondrial dysfunction, and oxidative stress (OS). Eventually causing brain energy metabolism disorders.

Several longitudinal studies show that glucose uptake slows down in the brain of AD patients (5–7). PET scans have shown that AD patients' brain areas, such as the posterior cingulate, temporal, parietal, and frontal lobes, especially in the hippocampus, have a near 10% glucose uptake deficit (8, 9). Some studies have indicated that Aβ plaques and intracellular neurofibrillary tangles in AD brains would decrease protein levels of GLUTs. The reduction of GLUTs will directly reduce the brain's glucose uptake, and may lead to the death of neurons as well as weakened neuroplasticity in certain brain areas (4, 7). Studies have confirmed that two toxic proteins, Aβ and over-phosphorylated tau protein, can also damage the level of glycolysis which causes the reduction of ATP production in the brain and impairs cognitive function (10).

Substantial epidemiological evidence suggests that insulin resistance (IR) is strongly associated with cognitive impairment (2, 11, 12). The central nervous system uses insulin and the insulin receptor as core factors to modulate glucose supply and energy homeostasis. They are critical for activating multiple signals like phosphatidylinositol 3-kinase (PI3-K), glycogen synthase kinase 3β (GSK-3β), and mitochondria for energy production (2). Brain IR will destroy brain energy homeostasis, impair glucose transport, and decrease glucose metabolism (13–17). Some studies have shown that brain IR can also increase the level of some inflammatory factors thereby activating microglia and astrocytes, causing glycolysis disorders and neuroinflammation (18, 19). Neuroinflammation will increase the energy demand in the brain and thereby further destroy the energy homeostasis.

Mitochondria, classically referred to as the “powerhouse” of the cell (20), play a crucial role in the production of ATP. More and more evidence indicates that mitochondrial dysfunction such as electronic transmission chain (ETC) and observed OXPHOS damage play essential roles in AD etiology and pathology (21, 22). Tau pathology mainly impairs complexes I and V which are the main components of OXPHOS in mitochondria (23, 24), and Aβ may reduce mitochondria redox capacity and decrease ATP production (25).

At the same time, IR and mitochondria dysfunction are the key factors in the production of reactive oxygen species (ROS) in the brain. The brain consumes about 25% of the body's total oxygen consumption, but has relatively few antioxidant enzymes compared to other organs (26). Brain OS occurs when ROS is greatly produced, which interferes with multiple cytosolic signaling pathways such as insulin/IGF1 signaling and MAPK signaling. Finally damaging the main energy metabolism of cells (2, 27).

Bibliometrics is the quantitative analysis that uses mathematical and statistical methods to quantitatively analyze and evaluate publications (28). Through bibliometric analysis of publications data, we can understand the current research focus, find highly cited publications, predict the future direction of research, and so on. CiteSpace and VOSviewer are Java web-based data processing and visualization applications (29), which offer good support in study reviews. And the Web of Science database is the key source of input data for bibliometric analysis. By extracting keywords from title recognition, abstracts, descriptors, and bibliographic documents, CiteSpace and VOSviewer will classify the boundary areas of the current study (30).

In this bibliometric study, we used CiteSpace and VOSviewer to evaluate publications about the mechanisms of brain energy metabolism disorders in mild cognitive impairment that have been listed in the Web of Science Core Collection database to analyze the current status, main contributors, study subjects, organizations, partnerships, and developments in research and emerging areas between 2000 and 2020.

## Data and Methods

### Data Collection

The search terms used to identify publications included: topic: (“Alzheimer disease” OR “Alzheimer's disease”) AND topic: (“brain energy” OR “energy metabolism” OR “brain metabolism” OR “brain energy metabolism”). The search was conducted for publications between 2000 and 2020, and the date of the retrieval was February 4, 2021. The data for bibliometric analysis from the Web of Science Core Collection search index included SCI-EXPANDED, SSCI, A&HCI, CPCI-S, CPCI-SSH, BKCI-S, BKCI-SSH, ESCI, CCR-EXPANDED, and IC.

### Inclusion Criteria

Inclusion criteria were: (1) Peer-reviewed published original articles on brain energy metabolism disorder mechanisms of AD, including basic and clinical research; (2) reviews on brain energy metabolism disorder mechanisms of AD; (3) articles published from 2000 to 2020 on Web of Science.

### Exclusion Criteria

Exclusion criteria were: (1) Publications involving plagiarism; (2) articles not officially published; (3) conference abstracts and proceedings and corrigendum documents; (4) unrelated articles; and (5) articles not retrieved from Web of Science or not in this time period.

### Analysis Methods and Tools

Clarivate Analytics, VOSviewer (1.6.16), and CiteSpace (5.7.R3) were used to perform this bibliometric analysis, by constructing a visual index graph to analyze the overall structure, clusters of measurement, and links between clusters and key points or pivot points. Clarivate Analytics was used for statistics.

VOSviewer was mainly used to generate cooperation network visualizations, an average annual publication year map, co-citation network visualizations, and density visualizations. In VOSviewer's cooperation network visualizations map and co-citation network visualizations map, different colors represent different clusters, and the lines between circles represent the cooperative relationship between different points. In the average annual publication year map, different colors correspond to different years. In the density visualizations map, the redder the color, the higher the density.

CiteSpace was mainly used to count centrality and generate cooperation network visualizations and citation burst year visualizations. In Citespace's cooperation network visualizations map, the outermost purple ring represents the centrality of each circle. The thicker the purple ring, the higher its centrality. The red ring represents a burst in a certain year. The line between the two circles represents the cooperative relationship between the two countries. The color of the line represents the closeness of the cooperation. The yellower the color, the closer the cooperation.

## Results and Discussion

### Bibliometric Analysis of Publication Outputs

**Figure 1:**
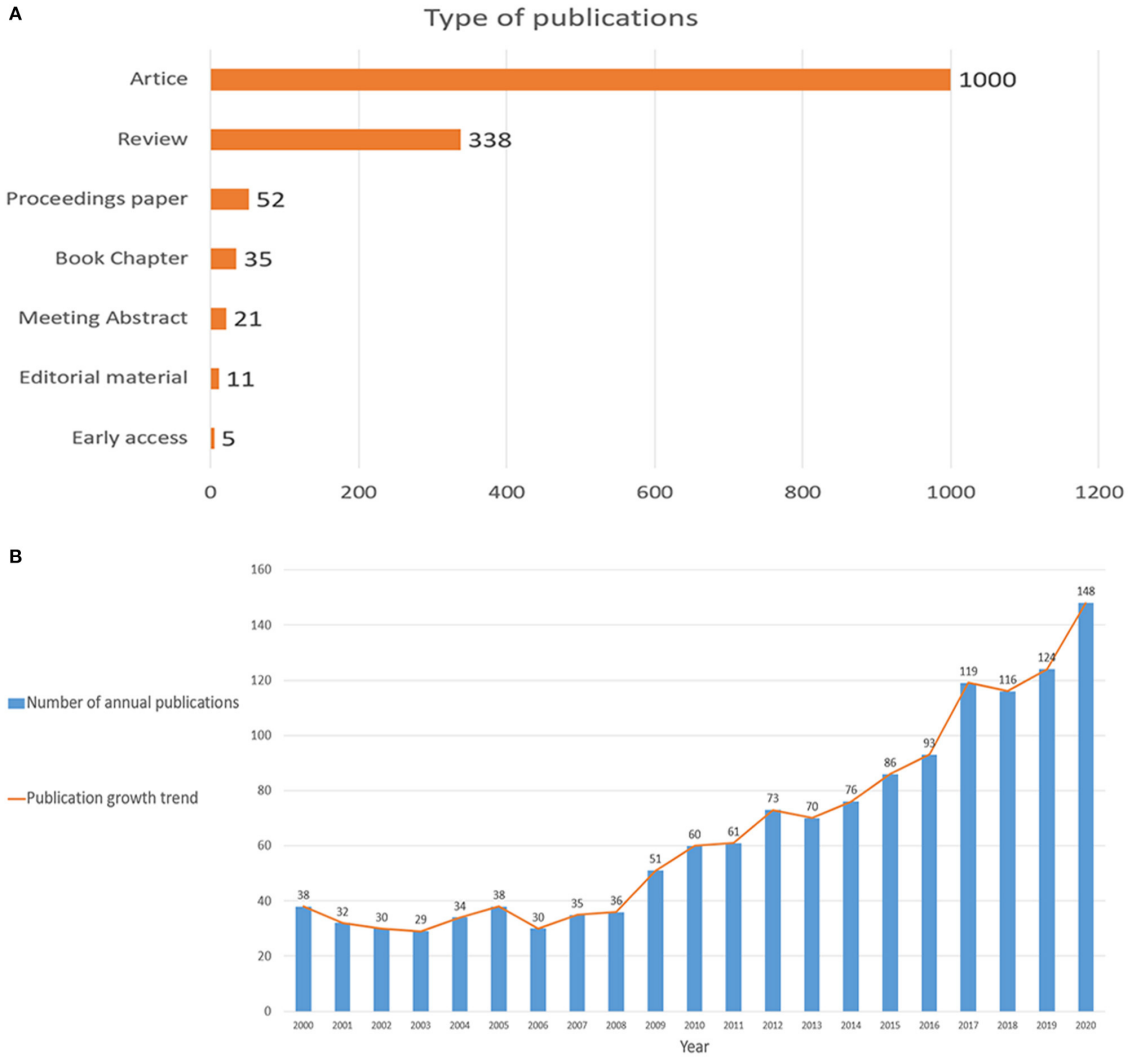
**(A)** Types of publications; **(B)** The combination chart of the number of annual publications.

This bibliometric analysis included a total of 1,379 publications from 2000 to 2020, including 1,000 research articles (72.5%), 338 reviews articles (24.5%), 52 proceedings papers (3.8%), 31 book chapters (2.5%), 21 meeting abstract (1.5%), 5 early accesses (0.4%), and 11 editorial materials (0.8%; Figure 1A). The language of most publications was English, accounting for 99.3%. The distribution of annual publications varied at different times, the first publication burst was from 2009 to 2016, when annual publications gradually rose from 30 to 100. The second burst was from 2017 to 2020, where the annual number of publications raised fiercely. In summary, the number of publications has been on the rise in recent years (Figure 1B).

### Bibliometric Analysis of Countries and Regions

**Figure 2:**
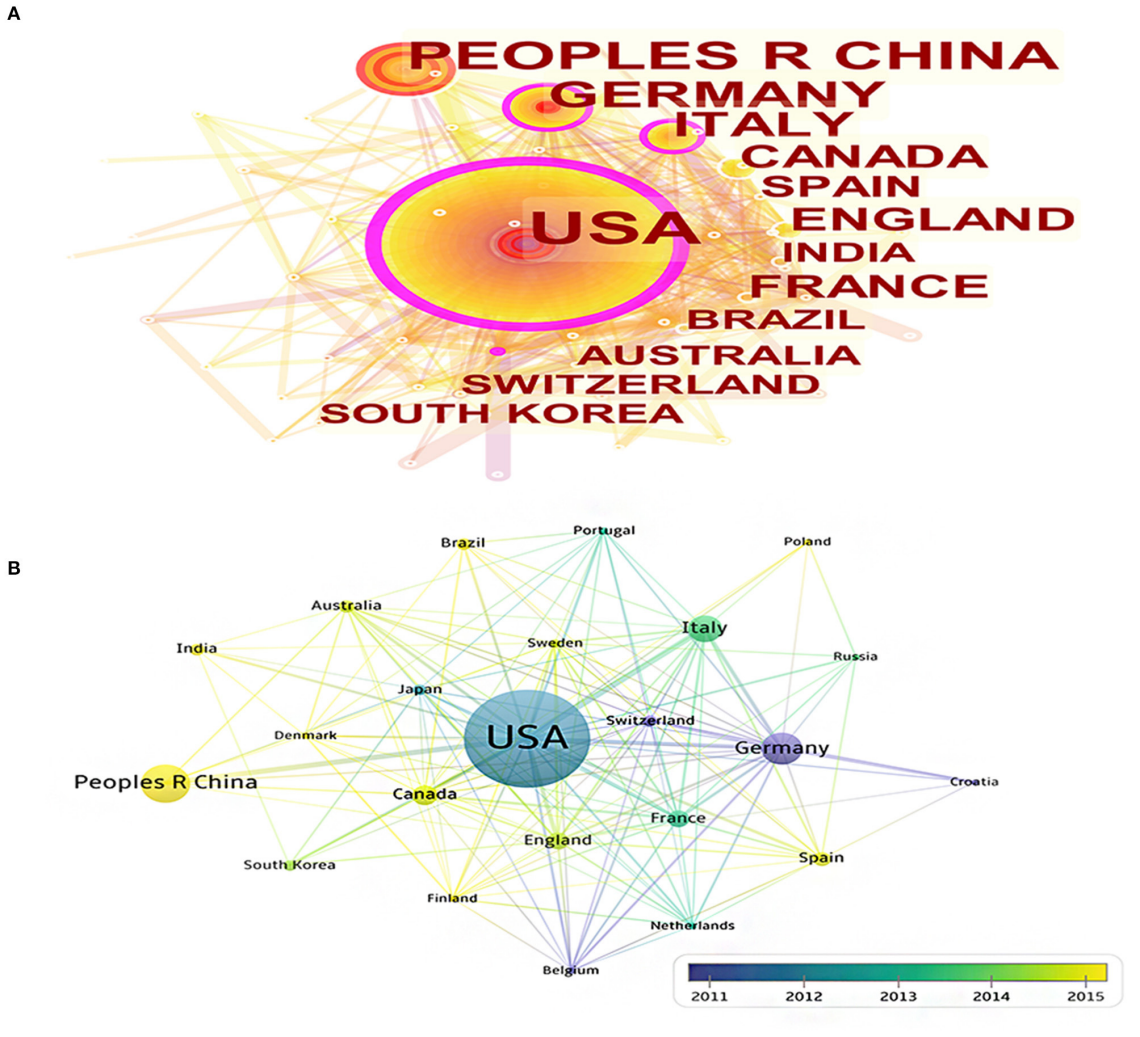
**(A)** The cooperation network visualizations map of countries and regions based on CiteSpace; **(B)** The average annual publication year map of countries and regions based on VOSviewer.

A total of 65 countries participated in the publication of mechanisms of brain energy metabolism disorders in AD. The countries and regions' cooperation network and density of the publications are shown on a map created by CiteSpace and VOSviewer (Figure 2A). Table 1 lists the top 10 most productive countries who published most of the publications. The USA, Germany, Italy, and Belgium had the most publications concentrated between 2012 and 2014, while publications from China, England, and India were mainly published after 2015 (Figure 2B). The USA published 565 publications and was cited 38,623 times which was the highest average citations (*n* = 68.36). From these two maps we can find that the USA had the highest centrality and density. There is no doubt that the USA is the most influential country in this field. China had the second largest number of publications (*n* = 181). But in terms of centrality (*n* = 0.01), number of citations (*n* = 4,540), and average citations times (*n* = 25.08), the quality of Chinese publications in this field is far below average. Germany, England, and Australia did not have a large number of publications, but in terms of average citation and density, the quality of their publications is very high.

**Table 1:** Countries and regions' publication number, centrality, and citations.

**Country**	**Counts**	**Centrality**	**Total citations**	**Average citations**
All Country	1,379	N/A	77,069	55.89
USA	565	0.33	38,623	68.36
China	181	0.01	4,540	25.08
Germany	147	0.19	9,405	63.98
Italy	118	0.16	6,094	51.64
Canada	87	0.04	3,177	36.51
France	69	0.07	3,097	44.88
England	68	0.10	4,929	72.49
Spain	56	0.04	1,380	24.64
Australia	48	0.03	3,987	83.06
India	45	0.03	1,837	40.82

### Bibliometric Analysis of Institutions

**Figure 3:**
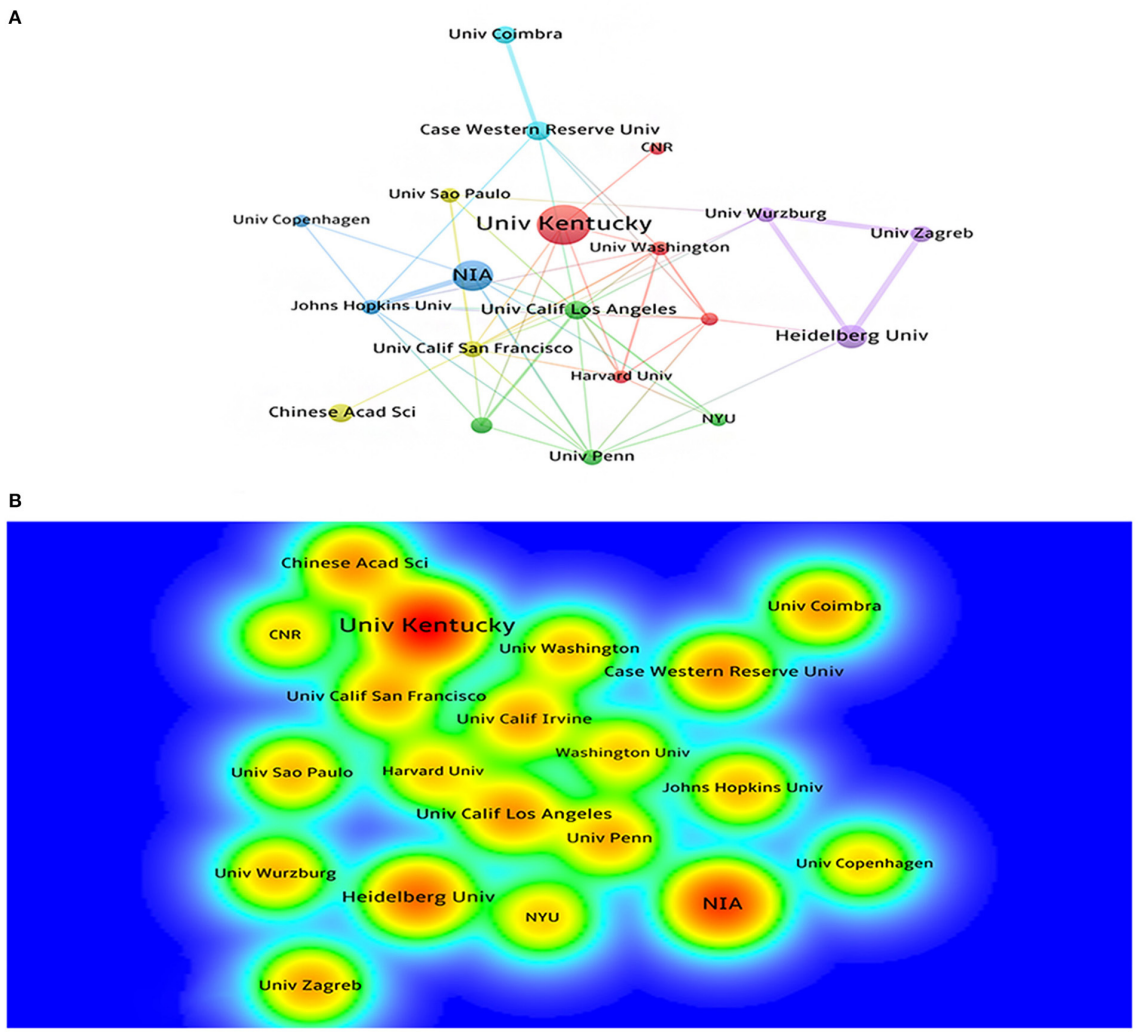
**(A)** The cooperation network visualization map of institutions based on CiteSpace; **(B)** The density visualization map of institutions based on VOSviewer.

A total of 525 institutions participated in the publication of mechanisms of brain energy metabolism disorders in AD. The most productive institutions can be found through the visualization maps (Figures 3A,B). Table 2 lists the top 10 most productive institutions, they published a total of 226 publications, accounting for 15% of the publications. There were six top institutions from the USA, with China, Germany, and Italy occupying the remaining four (Table 2). We can find that the initial research of most institutions started before 2003, Only Univ Kansas and CNR's initial research began in 2010. In terms of publication numbers and density, Univ Kentucky and NIA are the most productive institutions in this field. The cooperation among institutions in this field is relatively dispersed, and most of them form inherent cooperative groups, but the cooperation between the groups is not close enough.

**Table 2:** Institutions' publication number, country, and first publication year.

**Institution**	**Publication**	**Country**	**First publication year**
Univ Kentucky	46	USA	2003
NIA	40	USA	2000
Heidelberg Univ	23	Germany	2000
Case Western Reserve Univ	21	USA	2003
Cornell Univ	19	USA	2000
Univ Kansas	19	USA	2010
Chinese Acad Sci	18	China	2000
Univ Calif Los Angeles	14	USA	2001
Univ Wurzburg	13	Germany	2003
CNR	13	Italy	2010

### Bibliometric Analysis of Journals and Journals' Co-citations

**Figure 4:**
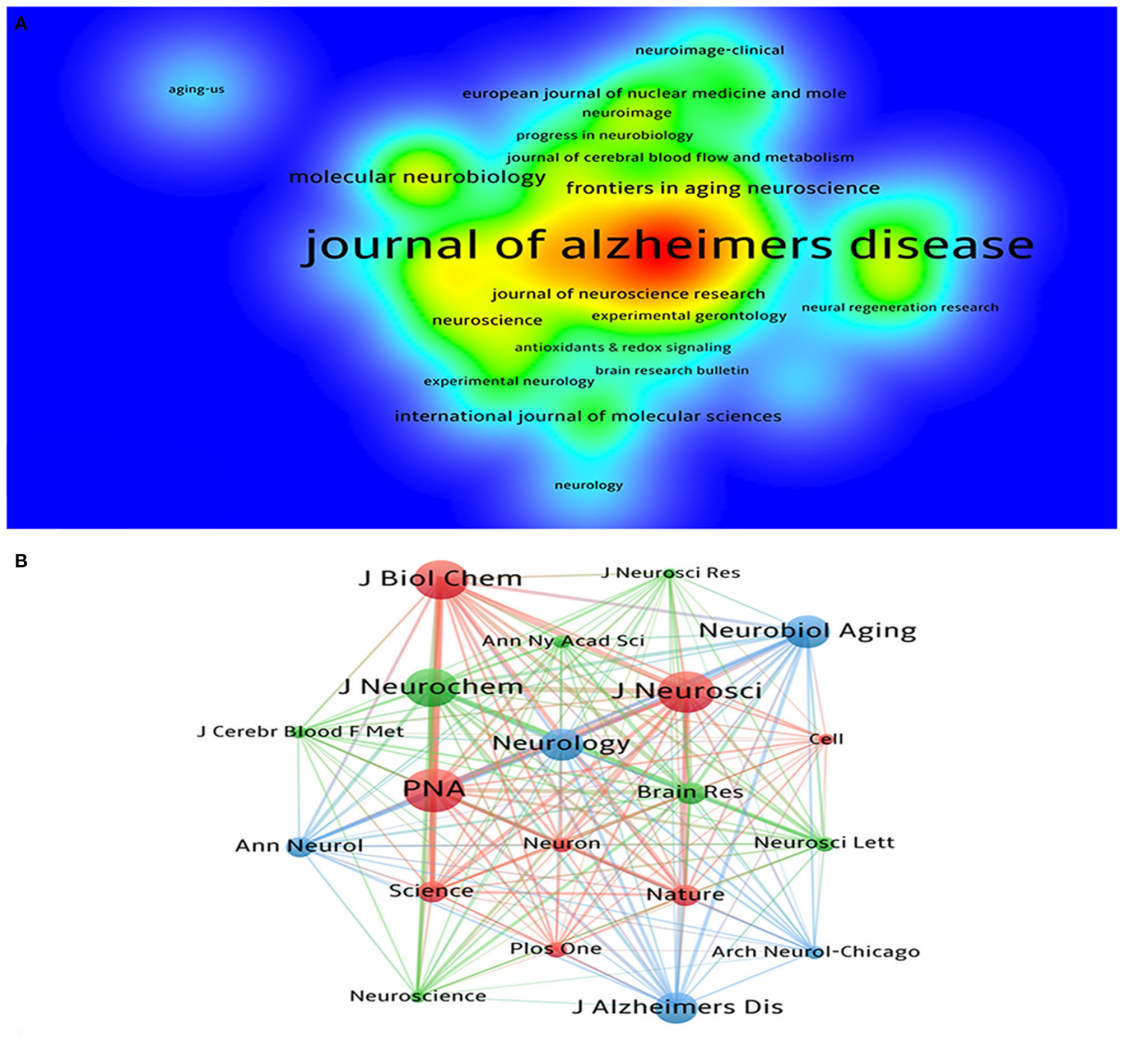
**(A)** The density visualization map of journals based on VOSviewer; **(B)** The co-citation network visualization map of journals based on VOSviewer.

A total of 481 journals participated in the publication of mechanisms of brain energy metabolism disorders in AD. Table 3 lists the top 5 most productive institutions, they published a total of 225 publications, accounting for 16.4% of the publications. The top five journals in the number of publications were the *Journal of Alzheimer's disease, Neurobiology of aging, Plos One, Frontiers in Aging Neuroscience*, and *Molecular Neurobiology* (Table 3). The top five co-cited journals were the *PNAS, Journal of Neurochemistry, Journal of Neuroscience, Journal of Biological Chemistry* and *Neurobiology of Aging* (Table 4). In terms of the number of publications, density, and citations, the *J*ournal of Alzheimer's disease and *Neurobiology of aging* are the most important journals in this field (Figure 4). In terms of influence, co-cited journals and journals with a high impact factor (IF) such as *PNAS, Journal of Neuroscience* and *Journal of Biological Chemistry* were also quite active in this field. Although they do not have many publications, they have a very high number of co-citations. This means that the quality of these journals' publications is far above average.

**Table 3:** Number of publications from the top five journals.

**Journal**	**Publications**	**Citations**	**IF (2019)**
Journal of Alzheimer's disease	98	5,194	3.909
Neurobiology of aging	41	2,243	4.347
Plos One	34	1,303	2.74
Molecular Neurobiology	28	989	4.5
Frontiers in Aging Neuroscience	24	800	4.364

**Table 4:** Top five co-cited journals.

**Journal**	**Publications**	**Co-citations**	**IF (2019)**
PNAS	8	3,599	9.412
Journal of Neurochemistry	20	3,209	4.066
Journal of Neuroscience	9	3,457	5.674
Journal of Biological Chemistry	9	3,258	4.106
Neurobiology of Aging	41	2,716	4.347

### Bibliometric Analysis of Authors and Authors' Co-citations

**Figure 5:**
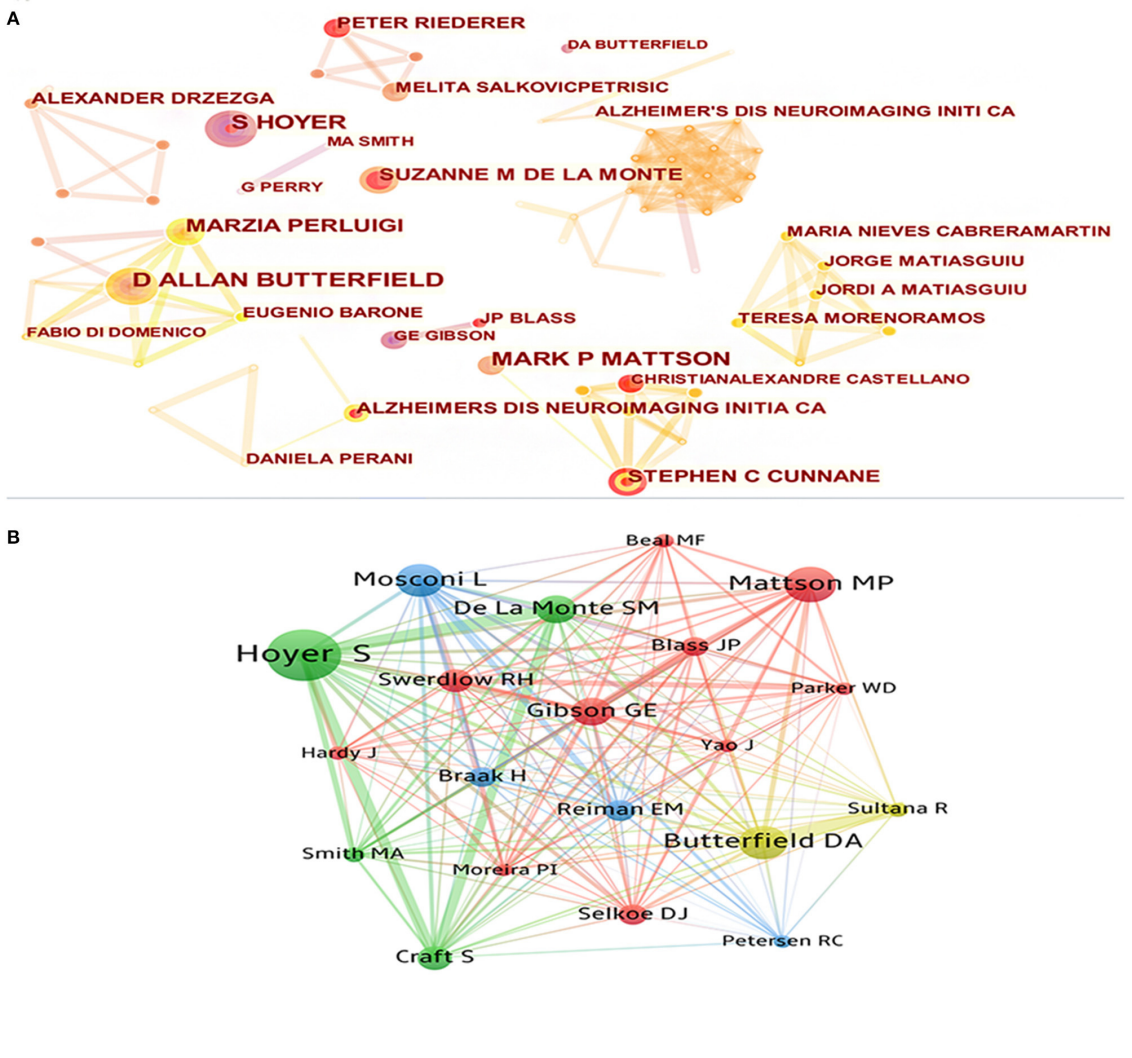
**(A)** The cooperation network visualization map of authors based on CiteSpace; **(B)** The co-citation network visualization map of authors based on VOSviewer.

Since 2000, a total of 6,449 researchers have participated in the publication of research in this field. Visualization maps can provide information on potential collaborators and can help researchers to establish collaborations (Figure 5). The top five cited authors were Butterfield DA, Mattson MP, Perluigi M, Stephen C, and Salkovic P. The top five co-cited authors were Hoyer S, Mattson MP, Butterfield DA, Mosconi L, and de la Monte SM (Table 5). In terms of the number of publications and number of citations, Mattson MP (h-index = 222) from Johns Hopkins University was undoubtedly the most influential and contributing author in this field. His research article “Beta-amyloid precursor protein metabolites and loss of neuronal Ca^2+^ homeostasis in Alzheimer's-disease” is his most cited article in this field, which discussed how an alternative app processing pathway affects brain energy metabolism by district Ca^2+^ homeostasis. Butterfield DA (h-index = 96) from University of Kentucky, De La Monte SM (h-index = 6) from Brown University, and Hoyer S (h-index = 46) from Heidelberg University also had great influence in this field. Butterfield DA's main research content was oxidative stress in AD. De La Monte SM mainly focused on understanding the role of insulin and insulin-like growth factor resistance in relation to neurodegeneration caused by AD. And Hoyer S's research was focused on cerebral metabolism in general, especially glucose metabolism.

**Table 5:** Top five cited authors and top five co-cited authors.

**Author**	**Publications**	**Citations**	**Author**	**Co-citations**	**Publications**
Butterfield DA	23	379	Hoyer S	585	11
Mattson MP	19	406	Mattson MP	406	19
Perluigi M	16	58	Butterfield DA	379	23
Stephen C	14	62	Mosconi L	374	3
Salkovic P	14	134	De La Monte SM	316	13

### Bibliometric Analysis of References

**Figure 6:**
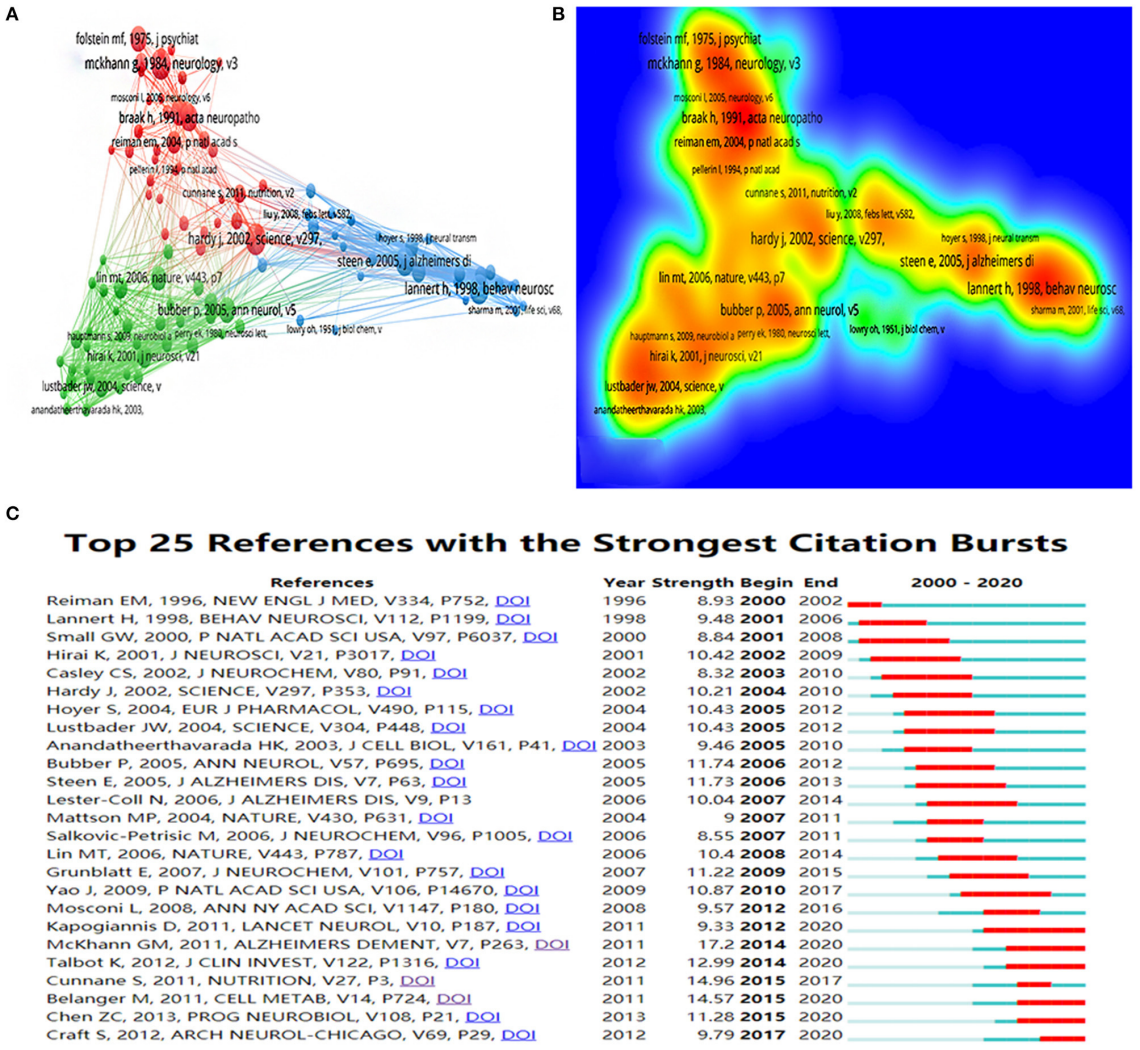
**(A)** The co-citation network visualization map of references based on VOSviewer; **(B)** The density visualization map of references based on VOSviewer; **(C)** Top 25 references with the strongest citation bursts based on CiteSpace, The red horizontal stripes represent the years with the most frequent publications. The green horizontal stripes represent the years with the most infrequent publications.

In the past 20 years, publications in this field have cited a total of 75,375 references, with an average of 55 citations per article. The top five cited references and top five centrality references are presented in Tables 6, 7. Figures 6A,B show the network and density of co-cited references. The top 25 references with the strongest citation bursts are presented in Figure 6C. According to the analysis of the co-citation counts and centrality among the 10 references, only one was an animal experiment, four were human experiments, two were reviews, one was a meta-analysis, and one was a recommendation. Four studies focused on insulin, insulin resistance, and glucose metabolism, four studies focused on mitochondrial disorders, and one study focused on oxidative stress. It is not difficult to find that insulin, mitochondria, and oxidative stress are key points in this field. According to the analysis of citation bursts, we can find that research on insulin and glucose metabolism in the brain is a hot topic in recent years. Among the publications whose bursts lasted until 2020, nearly half were about the insulin and glucose metabolism in the brain. These cited studies are landmark publications in this field, providing the foundation for future studies.

**Table 6:** Top five co-cited references.

**Rank**	**Co-cited (counts)**	**Reference**	**First author (year)**
1	63	The diagnosis of dementia due to Alzheimer's disease: recommendations from the National Institute on Aging-Alzheimer's Association workgroups on diagnostic guidelines for Alzheimer's disease	McKhann GM (2011) (31)
2	60	Mitochondrial bioenergetic deficit precedes Alzheimer's pathology in female mouse model of Alzheimer's disease	Yao J (2009) (32)
3	59	Brain fuel metabolism, aging, and Alzheimer's disease	Cunnane S (2011) (8)
4	58	Brain glucose hypometabolism and oxidative stress in preclinical Alzheimer's disease	Grünblatt E (2007) (33)
5	55	Demonstrated brain insulin resistance in Alzheimer's disease patients is associated with IGF-1 resistance, IRS-1 dysregulation, and cognitive decline	Talbot K (2012) (34)

**Table 7:** Top five references with the best centrality.

**Rank**	**Centrality**	**Reference**	**First author (year)**
1	0.16	Intranasal insulin therapy for Alzheimer disease and amnestic mild cognitive impairment: a pilot clinical trial	Craft S (2012) (35)
2	0.13	Mitochondrial damage in Alzheimer's disease varies with apolipoprotein E genotype	Gibson GE (2000) (36)
3	0.12	Accumulation of amyloid precursor protein in the mitochondrial import channels of human Alzheimer's disease brain is associated with mitochondrial dysfunction	Devi L (2006) (37)
4	0.12	Mitochondrial bioenergetic deficit precedes Alzheimer's pathology in female mouse model of Alzheimer's disease	Yao J (2009) (32)
5	0.11	Brain glucose metabolism in the early and specific diagnosis of Alzheimer's disease - FDG-PET studies in MCI and AD	Mosconi L (2005) (38)

### Bibliometric Analysis of Keywords

**Figure 7:**
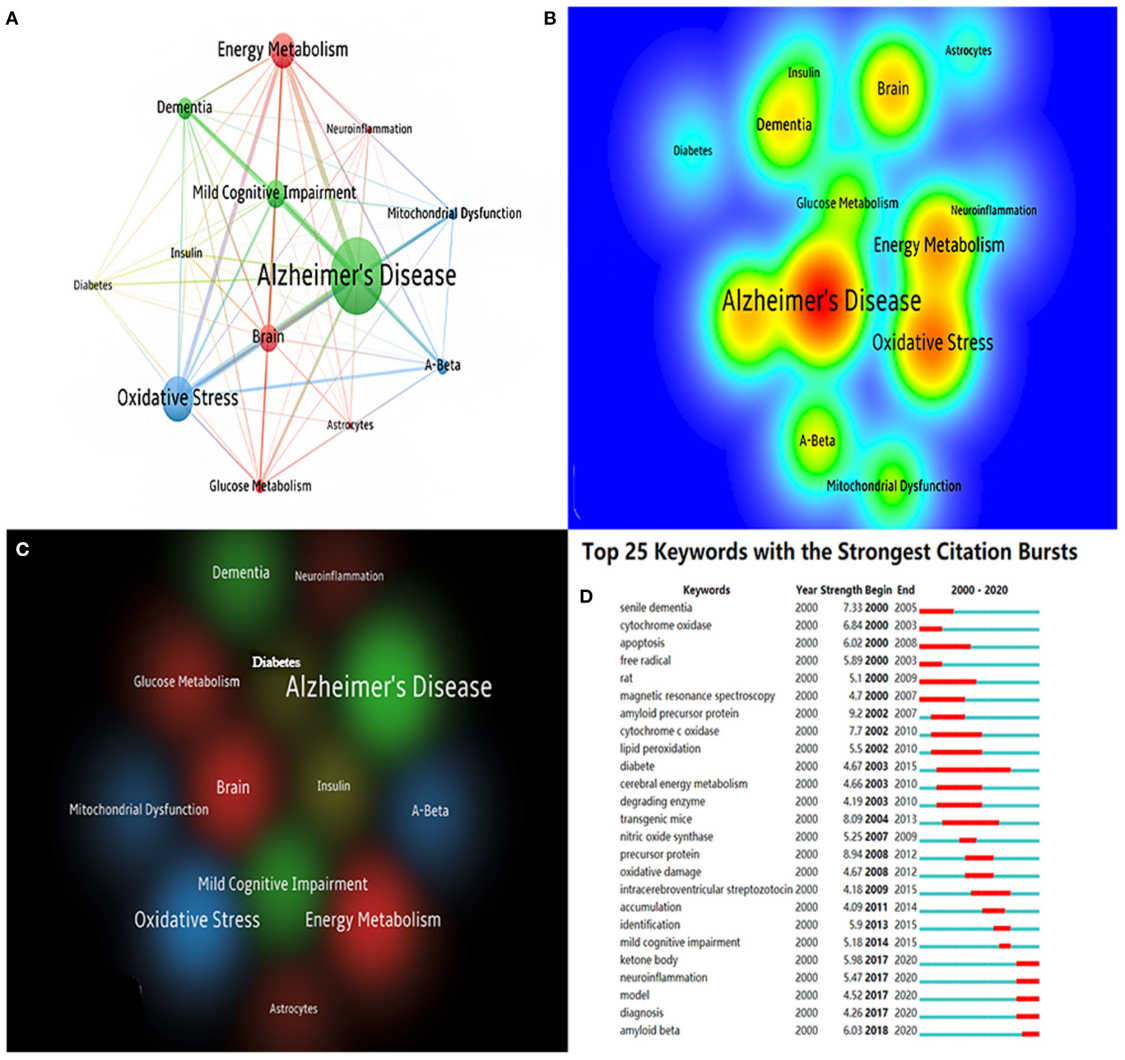
**(A)** The co-citation network visualization map of keywords based on VOSviewer; **(B)** The density visualization map of keywords based on VOSviewer; **(C)** The cluster visualization map of keywords based on VOSviewer; **(D)** The top 25 keywords with the strongest citation bursts based on CiteSpace, The red horizontal stripes represent the years with the most frequent keyword use. The green horizontal stripes represent the years with the most infrequent keyword use.

In a certain area of study, keyword co-occurrence may classify high-frequency keywords and investigate the strength of the relation between keywords by examining the co-occurrence of keywords in a broad number of publications. It can identify an academic field's internal structure and expose the discipline's research frontiers. The top 10 co-occurrence times of keywords are listed in Table 8. Figures 7A,B show the network and density of keywords. The top 25 keywords with the strongest citation bursts are presented in Figure 7D. There are four different keyword clusters generated in Figure 7C. There is a greater correlation between the keywords in each cluster. The green cluster contains some keywords such as Alzheimer's disease, mild cognitive impairment, and dementia. It is shown that brain energy metabolism disorders not only occur in the AD stage, but are more likely to occur in a much earlier stage such as the MCI stage. The red cluster contains some keywords such as neuroinflammation, glucose metabolism, brain, and energy metabolism. The blue cluster contains some keywords such as Aβ, oxidative stress, and mitochondrial dysfunction. The yellow cluster contains some keywords such as diabetes and insulin. These clusters show that Aβ, glucose metabolism disorders, insulin resistance, mitochondrial dysfunction, and oxidative stress are the current research focus in this field. Clarifying the relationship among them will be the key points of future research.

**Table 8:** Top 10 co-occurrence times of keywords.

**Rank**	**Counts**	**Keyword**	**Centrality**
1	617	Alzheimer's disease	0.09
2	368	Energy metabolism	0.06
3	362	Oxidative stress	0.03
4	224	Mild cognitive impairment	0.04
5	220	Brain	0.05
6	174	Dementia	0.05
7	164	Mitochondria	0.05
8	131	Aβ	0.03
9	110	Glucose metabolism	0.06
10	94	Mitochondrial dysfunction	0.06

From Figure 7D, we find that ketone, neuroinflammation, Aβ, glucose-related, and mitochondria-related were burst keywords in certain years. We found that in AD energy metabolism research, mitochondria, glucose, and diabetes were relatively early research directions, and research on ketone, neuroinflammation, and Aβ came relatively later than others.

## Conclusions

From the bibliometric analysis on the mechanisms of brain energy metabolism disorders in AD publications over the last 20 years, it was found that the number of articles published has gradually increased in recent years, which indicates that more and more researchers are starting to become involved in AD energy metabolism disorder research. Analyzed by country and institution, the USA and USA's institutions are undoubtedly the leaders in this field. China needs to improve the quality of its own publications while maintaining the number of publications. Connections between the various countries and organizations remain weak. Strengthening international collaboration is a top priority in this region.

Analyzed by journals and authors, we can find that the *J*ournal of Alzheimer's disease and *Neurobiology of aging* are the most important journals in this field, and some high IF journals also make a great contribution to the research in this field. Scientists led by Mattson MP, Butterfield DA, De La Monte SM, and Hoyer S are the backbone of this field. Analyzed by references, Talbot K (2012) (34), Yao J (2009) (32), Craft S (2012) (35), and Gibson GE (2000) (36) published articles with the highest number of citations and centrality, these publications are worthy of a deep study by rookie researchers in this field.

Analyzed by keywords, we can find that Aβ, tau, glucose metabolism disorders, insulin resistance, mitochondrial dysfunction, oxidative stress, ketone, and neuroinflammation are the key areas of research that reflect the world's central research directions over the past 20 years. Combined with recent clinical trials, we find that the current AD brain energy metabolism disorder research focuses on mitochondrial dysfunction, brain insulin resistance, and glucose metabolism disorders. Recent studies have shown that intranasal insulin can restore brain insulin function to treat early AD patients' brain energy metabolism (35, 39, 40). In a recent clinical trial, adults with mild Alzheimer's disease who obtained 20 or 40 IU of daily intranasal insulin increased their cognitive levels (40). In addition to the related treatment of drugs, many exercise therapies or non-invasive therapies have gradually become the focus of research in this field. In a clinical trial, the researchers found that daily transcranial direct current stimulation may maintain the regional cerebral metabolic rate for glucose in AD patients' middle/inferior temporal gyrus and delay their cognitive decline (41). Also, it has been proved that daily transcranial direct current stimulation can improve objective memory functioning and regional cerebral metabolic rate for glucose in multiple brains in mild cognitive impairment (42). Aerobic exercise is a hotspot of study in the area of AD treatment. Some studies show that aerobic exercise can improve brain energy metabolism in mild AD by increasing ketone uptake and utilization, maintaining brain glucose uptake, and improving insulin sensitivity (43, 44). Some animal experiments have confirmed that intermittent hypoxic exercise may become a new therapy for AD brain energy metabolism disorders by protecting and improving brain mitochondrial function (45, 46).

In conclusion, brain energy disorders play a central role in the development of AD. Many patients have already experienced energy shortage in the brain when there are no or only mild symptoms. In the future, how to cure or avoid brain energy metabolism disorders may become a major research topic in the prevention of AD. This paper uses bibliometric analysis to demonstrate the state of study in this area over the last 20 years. This will aid researchers in this field in enhancing international and regional collaboration, deepen the understanding of popular research in this field as well as help grasp and analyze potential research hotspots and directions in this field.

## Data Availability Statement

The original contributions presented in the study are included in the article/supplementary material, further inquiries can be directed to the corresponding author/s.

## Author Contributions

MC, YS, and LZ designed the study and conceived the article. Y-HD interpreted the data and wrote the article. QW collected and analyzed the data. L-CL checked the data. R-YY and L-YW revised the article. All authors contributed toward data analysis and drafting and critically revised and approved the final version of the paper.

## Conflict of Interest

The authors declare that the research was conducted in the absence of any commercial or financial relationships that could be construed as a potential conflict of interest.
